# Current achievements and future prospects in the genetic breeding of chrysanthemum: a review

**DOI:** 10.1038/s41438-019-0193-8

**Published:** 2019-10-01

**Authors:** Jiangshuo Su, Jiafu Jiang, Fei Zhang, Ye Liu, Lian Ding, Sumei Chen, Fadi Chen

**Affiliations:** State Key Laboratory of Crop Genetics and Germplasm Enhancement, Key Laboratory of Landscaping, Ministry of Agriculture and Rural Affairs, College of Horticulture, Nanjing Agricultural University, 210095 Nanjing, China

**Keywords:** Plant breeding, Genetic techniques, Plant genetics

## Abstract

Chrysanthemum (*Chrysanthemum morifolium* Ramat.) is a leading flower with applied value worldwide. Developing new chrysanthemum cultivars with novel characteristics such as new flower colors and shapes, plant architectures, flowering times, postharvest quality, and biotic and abiotic stress tolerance in a time- and cost-efficient manner is the ultimate goal for breeders. Various breeding strategies have been employed to improve the aforementioned traits, ranging from conventional techniques, including crossbreeding and mutation breeding, to a series of molecular breeding methods, including transgenic technology, genome editing, and marker-assisted selection (MAS). In addition, the recent extensive advances in high-throughput technologies, especially genomics, transcriptomics, proteomics, metabolomics, and microbiomics, which are collectively referred to as omics platforms, have led to the collection of substantial amounts of data. Integration of these omics data with phenotypic information will enable the identification of genes/pathways responsible for important traits. Several attempts have been made to use emerging molecular and omics methods with the aim of accelerating the breeding of chrysanthemum. However, applying the findings of such studies to practical chrysanthemum breeding remains a considerable challenge, primarily due to the high heterozygosity and polyploidy of the species. This review summarizes the recent achievements in conventional and modern molecular breeding methods and emerging omics technologies and discusses their future applications for improving the agronomic and horticultural characteristics of chrysanthemum.

## Introduction

Chrysanthemum (*Chrysanthemum morifolium* Ramat.) belongs to the Asteraceae family and is one of the most economically important and favored floricultural crops, ranking second in the cut flower trade after rose^1^. Chrysanthemum has a long history of cultivation; it was first cultivated in China as a herb in approximately the 15^th^ century BC and was then successively introduced to Japan, Europe, and the United States^2^. The ancestry of modern chrysanthemum is still uncertain, but the plant is thought to have emerged mainly as a result of long-term artificial selection of variants of several wild species, including *C. vestitum* (2n = 54), *C. indicum* (2n = 18, 36), *C. lavandulifolium* (2n = 18), *C. nankingense* (2n = 18), and *C. zawadskii* (2n = 54)^3–5^. Cultivated chrysanthemum is a complex hexaploid that also exhibits aneuploidy, in which chromosome numbers vary from 47 to 67^6,7^. However, a chromosome number of 54 is the most frequent and stable conformation (2n = 6x = 54).

**Fig. 1:**
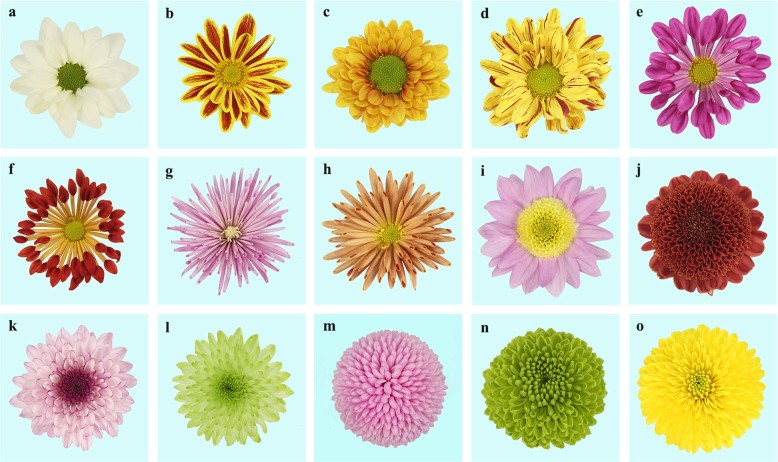
The abundant genetic variations of flower color and shape in chrysanthemum. **a**, **b** Single type; **c**, **d** double type; **e**, **f** windmill type; **g**, **h** pine needle type; **i**, **j** anemone type; **k**, **l** incurve type; **m**–**o** pompon type

Genetic resources are crucial for all plant-breeding programs. Fortunately, rich chrysanthemum genetic resources with abundant natural phenotypic variation are available. The capitulum, a typical ornamental part of the chrysanthemum flower, is composed of two types of morphologically distinct florets. The marginal ray florets are naturally male sterile and exhibit various colors that are specific to each cultivar, while the central disc florets are hermaphroditic with drab yellow or green colors. Different combinations of floret number, petal size, and floral organ fusion lead to various flower shapes, such as the single, double, windmill, pine needle, anemone, incurve, and pompon types (Fig. 1). According to the growth habit and cultivation type, chrysanthemums are mainly classified into the traditional type, cut chrysanthemum types (including the spray and disbud-cut types), potting type, and groundcover type^8,9^. The species is widely cultivated for the production of cut flowers and used in gardening, potted plants, and ornamental landscaping^10^. Chrysanthemum flowers contain significant amounts of nutritive and biologically active components^11^. Hence, chrysanthemums are also used in the medical, food, and beverage industries. In addition to its great economic and ornamental value, chrysanthemum holds special cultural significance. It has been honored as one of the ‘four gentlemen among flowers’, together with plum blossom, orchid, and bamboo, in Chinese classic literature, and it is included in the heraldry of the Japanese Imperial Family as an emblem of courage.

The market demands for chrysanthemum increase annually, forcing scientists and breeders to create new cultivars with novel appearances and improved stress tolerance and quality attributes. Conventional breeding approaches, including crossbreeding and mutation breeding, are the most common approaches used to develop new chrysanthemum cultivars. However, in classical approaches, breeders mainly use phenotypes to select superior progeny or mutants, a process that is often laborious and ineffective, especially for traits with significant genotype × environment (G × E) interactions^12,13^. In addition, some desired traits, such as a blue hue, cannot be produced by hybridization and mutation due to the limitations and drawbacks of these techniques. In recent years, transgenic molecular breeding has been extensively employed by introducing foreign genes into chrysanthemum using *Agrobacterium*-mediated transformation and biolistic transformation and has led to considerable progress in horticultural character improvement^1,14,15^. In addition, the rapid development of DNA-based molecular marker techniques offers plant breeders a new opportunity to employ molecular marker-assisted selection (MAS) in breeding, which allows the indirect selection of target traits without regard to environmental factors or plant growth phases and shows great potential to increase the effectiveness of plant breeding^16,17^.

In addition, the advent and improvement of next-generation sequencing (NGS) technology has accelerated the generation of multiomic data at the DNA, RNA, protein, and metabolite levels at an unprecedented rate, leading to a new era of ‘big biological data’^18^. Organizing and integrating these big data and linking them to particular phenotypes will contribute significantly to broadening and deepening our understanding of the molecular mechanisms and genetics of living organisms. Multiomic technologies have successfully assisted in the breeding programs of major agricultural crops^19–21^. However, the development and application of sophisticated omic techniques in chrysanthemum is a daunting task and has lagged behind the progress made in model plants and agricultural crops; this circumstance may be primarily due to the high heterozygosity and polyploidy of chrysanthemum or a lack of suitable genomic information. This review summarizes the merits and drawbacks of conventional breeding. We mainly focus on recent advances and challenges in the application of molecular and multiomic technologies in chrysanthemum and discuss their future prospects for accelerating breeding programs.

## Conventional breeding

### Crossbreeding

Chrysanthemum is a highly heterozygous plant that shows inbreeding depression and self-incompatibility; as a result, conventional crossbreeding is a powerful method for developing modern chrysanthemum cultivars. Many commercial cultivars with desirable traits, such as the Mammoth series garden chrysanthemum ‘Lavender Daisy’, have been developed via crossbreeding^22^. Moreover, many important wild species harbor a variety of favorable resistance genes that can be introduced into florist chrysanthemums via intergeneric or interspecific breeding^1,8^. Crossbreeding is simple and effective, and the F_1_ progeny derived from two parents with contrasting target traits generally exhibit a wide phenotypic variation in chrysanthemum. However, there are several factors that must be taken into consideration when crossbreeding chrysanthemum, such as the fertility of a certain cross combination, qualitative analysis of the target traits, and superior hybrid progeny selection. In addition, hybrid progeny with the desired traits often also carry other less preferred traits that require several generations for removal through backcrossing, a process that greatly increases the cost and labor associated with crossbreeding. To make better use of the crossbreeding technique in chrysanthemum, Su et al.^23^ used molecular markers associated with target traits. An effective mathematical analysis method was proposed that combined the analytic hierarchy process (AHP) and gray relational analysis (GRA) to evaluate progress in breeding new early-flowering multiflora chrysanthemum cultivars and to define the flowering times of cultivated plants^24^.

### Mutation breeding

Mutation breeding is a useful strategy in vegetatively propagated ornamental plants that has been widely exploited to modify one or a few traits in an outstanding cultivar without altering its other phenotypic traits^25^. Normally, mutations occur spontaneously, although they can be induced by physical and chemical methods. For the past 30 years, the mutation technique has successfully produced a large number of new chrysanthemum varieties that have been commercialized. For example, members of the ‘Anna’ chrysanthemum series were produced from natural mutations. In addition, numerous chrysanthemum cultivars with novel traits, especially new flower colors and shapes, have been produced by X-radiation, gamma radiation, and ion-beam irradiation^1,26,27^. More recently, microwave radiation, which is a form of electromagnetic radiation, has been used to induce genetic and phenotypic variations (novel flower shape and color, increased inflorescence diameter, and prolongation of bud coloration) in chrysanthemum^28^. The advantage of mutation breeding is that the high heterozygosity of chrysanthemum can increase the apparent mutation rate and produce many excellent mutation types in a short period of time. However, the primary limitations of this type of breeding are that mutations occur unpredictably throughout the genome, and often, only a single change is produced. Moreover, the feasibility of mutation breeding depends on several factors, such as the selection of a suitable genotype, explant type, induction mutation method, and optimal dose^29^. For example, plants with pink flower color are regarded as the best starting material to produce other flower colors using ionizing radiation^30^. Thus, it is difficult to obtain a mutant with multiple desired characteristics. To overcome these disadvantages, a large mutant population, large field trials, extensive labor, and an appropriate genotype are required to obtain a promising mutant. Combining crossbreeding, which can easily generate mixtures of traits, with mutation breeding would also be helpful. In addition, chimerism may be an important phenomenon with respect to the generation of genetic variation, especially in the earlier stages of mutation-breeding programs^31^. It is presumed that most spontaneous and artificial mutants of chrysanthemum are periclinal chimeras in which the mutant genes exist in only the outermost L1 layer, which is genetically different from the others. To resolve the issue of chimerism, a combination of tissue culture and irradiation has been preliminarily shown to be the most efficient method for avoiding chimerism in chrysanthemum^32^. More intensive research on periclinal chimerism is needed in the future.

## Molecular breeding

### Transgenic breeding

Transgenic technology can be used to transfer genes to a host plant from any source and to repress or enhance gene expression in a programmable manner. Compared with conventional breeding strategies, the transgenic method possesses greater prospective potential for producing innovative phenotypes. Transgenic ornamental plants are becoming beneficial for both growers and consumers due to their novel appearances and enhanced stress tolerance without presenting food safety concerns, in contrast to fruits and vegetables^31^. Transgenic technology has become an important means of breeding new chrysanthemum cultivars and has led to great achievements related to floral attributes, plant architecture, postharvest flower longevity, and biotic and abiotic stress tolerance, as reviewed by Teixeira da Silva et al.^1^ and Cheng et al.^33^ Hence, this review describes only the most recent reports of several useful traits that have been introduced into chrysanthemum.

#### Abiotic and biotic stress tolerance

The industrialization of chrysanthemum is limited by several abiotic and biotic stresses, including drought, salt, extreme temperature, and insect pests, and the development of new cultivars with increased tolerance has always been a goal of breeders. Li et al.^34^ observed that overexpressing *CmHSFA4* increased the salt tolerance of chrysanthemum by limiting Na^+^ accumulation and maintaining the K^+^ concentration, and this tendency was consistent with that observed for the ion transporters *CmSOS1* and *CmHKT2*. The chrysanthemum WRKY transcription factor (TF) genes have been implicated in different cellular processes involved in stress responses such as salt^35–37^, drought^38,39^, disease^40^, and aphid resistance^41^. Du et al.^42^ cloned and functionally characterized *CmDREB6*, which was classified into the DREB A-6 subgroup, and found that overexpression of *CmDREB6* increased the heat tolerance of chrysanthemum by affecting genes involved in the heat-shock response and reactive oxygen species (ROS) homeostasis. Qi et al.^43^ isolated the chrysanthemum gene *CmCPL1*, which encodes RNAPII CTD phosphatase-like 1, and showed that overexpression or knockdown of *CmCPL1* increased or decreased the heat tolerance of chrysanthemum, respectively. Overexpression of the *C. lavandulifolium* gene *ClCBF1* in the chrysanthemum cv. ‘White Snow’ improved its salt and drought tolerance^44^.

#### Plant growth and development

Growth and development, especially floral organ development, are important characteristics of flowering plants. Floret number and petal size define flower shape in chrysanthemum. Six *CmCYC*_2_ genes specifically expressed in the petals of ray florets were identified in *C. lavandulifolium* by Huang et al.^45^ Overexpression of *CmCYC*_2_*c* significantly increased flower number and the length of ray florets but was insufficient to completely change the flower shape, indicating that flower shape might be a complex trait with polygenic inheritance in chrysanthemum. The TCP gene family plays a significant role in plant growth and development. Wang et al.^46^ isolated *CmTCP20*, which belongs to the PCF group in Class I of the TCP TF family, from chrysanthemum and found that it may be responsible for petal elongation growth. The root system is the main organ through which plants absorb water and nutrients. Sun et al.^47^ observed that overexpression of *CmANR1*, a MADS-box TF gene, promoted adventitious root and lateral root development in chrysanthemum. Further study indicated that *CmANR1* function directly regulates the auxin transport gene *CmPIN2*. The ability to manipulate plant architecture increases the ornamental value, and hence, the marketability of a commercial species. Shoot branching directly determines plant architecture and is a key determinant of plant shape that can improve plants’ capacity to adapt to environmental stresses. Enhancement of branching is possible by introducing *IPT1*^48^. *D14*^49^ and *D27*^50^ were found to play diverse roles in the regulation of shoot branching affected by hormonal and environmental factors in chrysanthemum, and Nie et al.^51^ isolated *CmERF053* from chrysanthemum, which was found to possess the new function of regulating shoot branching and lateral root development, in addition to affecting drought resistance.

#### Flowering time

Flowering time is the main determinant of successful commercial plants, and the development of early-flowering cultivars helps meet consumers’ needs by allowing plants to bear more flowers or be produced in sufficient numbers for the celebration of particular festivals. Yang et al.^52^ created transgenic RNA interference (RNAi)-suppressed chrysanthemum plants that flowered ~20 d earlier than *Cm-BBX24-*overexpressing and wild plants under long-day conditions. The transgenic *Cm-BBX24* plants also responded to salt and drought stresses, possibly due to changes in gibberellic acid (GA) biosynthesis. The mechanisms by which plant age regulates flowering remain incompletely understood. Wei et al.^53^ showed that age-dependent regulation of SPL TFs by *miR156* influences flowering by controlling *CmNF-YB8* expression in chrysanthemum. More recently, overexpression of *CmERF110* in transgenic Arabidopsis accelerated flowering by ~7 d compared with nontransgenic plants^54^. Moreover, several flowering-time-related genes, such as *TFL*^55,56^, *FTL*^57^, *CPD* and *GA20*^58^, *MET1*^59^, and their homologs, have been isolated from chrysanthemum.

#### Flower color

Flower color is a major objective of ornamental plant breeding due to its strong influence on consumer choice. Although diverse flower colors have been observed in chrysanthemum during its long history of cultivation, violet and blue colors are lacking due to a deficiency in flavonoid 3′5′-hydroxylase (F3′5′H) activity^60^. F3′5′H is a key enzyme for delphinidin biosynthesis in most blue-hued flowers. In the past, transgenic chrysanthemums with a violet ray-petal color were produced by expressing a heterologous F3′5′H gene under the control of ray-petal-specific promoters^61,62^. However, several internal and external factors, such as vacuolar pH, metal ions, and coexisting colorless compounds, also affect the formation of blue pigments or flowers. More recently, Noda et al.^63^ successfully generated true blue chrysanthemum flowers by introducing *CtA3*′*5*′*GT*, encoding UDP-glucose:anthocyanin 3′,5′-O-glucosyltransferase from *Clitoria ternatea*, and *CamF3*′*5*′*H*, encoding F3′5′H from *Campanula medium*. The delphinidin-based anthocyanins synthesized in the ray petals of the transgenic plants were glycosylated at the *3*′*5*′ positions and displayed bluer color via intermolecular association with flavone glucosides.

#### Genome editing

Recently, genome editing has been used as a vital tool for functional genomics and biotechnology research and has become available as a precision-breeding approach for modifying traits in plant species^64^. Genome-editing techniques theoretically allow researchers to introduce mutations into any targeted genomic sequence, and mutations in genome-edited plants are heritable. To date, a few reports have demonstrated successful genome editing for flower color in ornamental plants^65,66^ by using the CRISPR/Cas9 method. However, the introduction of mutations into polyploids, especially in nonmodel species, is rather difficult due to the double-strand breaks and repair events that occur independently. Moreover, the possibility of a mutation occurring decreases with the increase in target site number in polyploids^67^. Kishi-Kaboshi et al.^68^ first attempted to use multicopy transgenes instead of endogenous genes as targets for genome editing in chrysanthemum. The resultant transgenic chrysanthemum possessed over five copies of the yellowish-green fluorescent protein gene (*CpYGFP*) from *Chiridius poppei*, allowing the visualization of gene-editing progress. Two single-guide RNAs (sgRNAs) were used to target different positions in the *CpYGFP* gene, and transgenic lines with mutated *CpYGFP* genes were acquired, showing that axillary buds and callus regeneration contribute to mutation. The researchers suggested that by introducing an expression cassette that encodes Cas9 and sgRNA, complete gene mutations could be obtained through continuous culture or cuttings in chrysanthemum. These findings shed light on chrysanthemum genome editing and provide breeders with new tools for precise breeding.

#### Remarks on transgenic breeding

Overall, several useful traits have been introduced into chrysanthemum by transgenic technology, and the production of transgenic plants has great potential to reduce production costs and improve stress resistance, flower quality, and hence, the commercial value of chrysanthemum. The development of new cultivars with excellent comprehensive characteristics is a major target of chrysanthemum-breeding programs. However, most of the relevant published reports involve single-gene transfer and lack appropriate conversion systems. Hence, establishing effective transformation systems for more receptor plants, improving conversion efficiency, and realizing multigene cotransformation are three major challenges in chrysanthemum transgenic breeding. The modern horticultural industry is beginning to take safe production seriously; although no food safety problems are associated with transgenic ornamentals, they may impact the environment, and transgenic technology is strictly limited, and thus, difficult to exploit by breeders in many countries^55^. Therefore, the development and utilization of environmentally friendly production systems to improve the safety of chrysanthemum molecular breeding will be a focus of future transgenic technology research. Genome-editing technology is a precision-breeding strategy regardless of legal concerns, but additional techniques are required for isolating target genes and generating plants with multiple target mutations in polyploid chrysanthemum^68^.

### Marker-assisted selection

The MAS breeding of ornamental plants is relatively new. Several molecular marker types ranging from conventional gel-based random amplified polymorphic DNA (RAPD), amplified fragment length polymorphism (AFLP), sequence-related amplified polymorphism (SRAP), and simple sequence repeat (SSR) markers to more current sequence-based single-nucleotide polymorphism (SNP) markers have been applied to identify markers that are significantly associated with target traits in chrysanthemum. The recent progress of genetic studies based on molecular markers in chrysanthemum is summarized in Table 1.

**Table 1:** Recent published genetic analyses in chrysanthemum

Population	Population size	Marker type	Marker number	Study objective	Methodology	Reference
‘Yuhualuoying’ × ‘Aoyunhanxiao’	142	RAPD, ISSR, and AFLP	336	Linkage map construction	Map construction	^74^
‘Yuhualuoying’ × ‘Aoyunhanxiao’	142	SRAP	675	Inflorescence traits	QTL mapping	^75^
‘Yuhualuoying’ × ‘Aoyunhanxiao’	142	SRAP	675	Plant architectural traits	QTL mapping	^76^
‘Yuhualuoying’ × ‘Aoyunhanxiao’	142	SRAP	675	Leaf traits	QTL mapping	^77^
‘Yuhualuoying’ × ‘Aoyunhanxiao’	142	SRAP	346	Flowering time	One-way ANOVA	^78^
‘Yuhualuoying’ × ‘Aoyunhanxiao’	142	SRAP	675	Flowering time	QTL mapping	^79^
‘Han 2’ × ‘Nannong Gongfen’	133	SRAP, SSR, and SCoT	262	Aphid resistance	One-way ANOVA, QTL mapping, and BSA	^80^
‘QX145’ × ‘Nannongyinshan’	92	SRAP and SSR	234	Branching traits	QTL mapping	^81^
‘QX053’ × ‘Nanong Jingyan’	160	SRAP, SSR, and SCoT	497	Inflorescence traits	One-way ANOVA and QTL mapping	^82^
‘Nannong Xuefeng’ × ‘Monalisa’	162	SRAP and SSR	502	Waterlogging tolerance	QTL mapping	^83^
‘Kitam’ × ‘Relinda’	86/160	AFLP	1000/327	Branching traits	Candidate gene-based association study/one-way ANOVA, and BSA	^101^
‘Puma White’ × ‘Dancer’	94	AFLP	1779	White rust	BSA	^106^
Natural population	159	SRAP, SSR, and SCoT	707	Plant architecture and inflorescence	GWAS	^95^
Natural population	100	SRAP, SSR, and SCoT	707	Waterlogging tolerance	GWAS	^96^
Natural population	80	SRAP, SSR, and SCoT	707	Aphid resistance	GWAS	^97^
Natural population	159	SRAP, SSR, and SCoT	707	Drought tolerance	GWAS	^98^
DB36451×DB39287	406	SNP	183,000	Flowering time and inflorescence traits	QTL mapping	^92^
Natural population	199	SNP	92,830	Cultivated type and inflorescence traits	GWAS	^9^
Natural population	88	SNP	92,811	Waterlogging tolerance	GWAS	^99^
Natural population	107	SNP	92,617	Inflorescence traits	GWAS	^100^

#### Linkage maps and QTL mapping

A linkage map provides a basis for the identification of genomic regions related to traits of interest and the necessary infrastructure for MAS breeding^69,70^. Quantitative trait locus (QTL) mapping is a conventional tool for identifying the genes that control a trait, and it is useful for genome-wide scanning for QTL detection based on a linkage map in plants^71^. Constructing a genetic linkage map is more difficult in polyploids than in diploids due to the unknown linkage phases of the marker alleles in the former; thus, linkage map construction in polyploids requires specialized methods^72^. Although earlier cytological studies revealed polysomic inheritance in chrysanthemum, cultivated chrysanthemums have long been assumed to exhibit disomic inheritance because of their presumed multispecies origin and the occurrence of bivalent chromosomal pairing^72,73^. Generally, linkage maps in chrysanthemum are most efficiently constructed according to the ‘double pseudotestcross’ mapping strategy in an F_1_ population. The first preliminary linkage map of chrysanthemum was produced by Zhang et al.^74^ from a biparental cross using a combination of RAPD, AFLP, and inter simple sequence repeat (ISSR) markers. Their study failed to produce a referenceable integrated map, and only six pairs of homologous linkage groups (LGs) based on the common intercross markers of the two parental maps were identified. Furthermore, the authors constructed other maps for the same population using SRAP markers, with an average map distance of 6.9 cM for the female map and 6.6 cM for the male map^75^. Subsequently, the SRAP-based maps were used to detect QTLs for inflorescence-related traits^75^, plant architectural traits^76^, leaf traits^77^, and flowering time and duration^78,79^. In the past 5 years, several F_1_ mapping populations have been generated with the aim of genetically localizing different horticultural traits, i.e., aphid resistance^80^, branching traits^81^, traits related to anemone-type flowers^82^, and waterlogging tolerance^83^, by linkage map construction and QTL mapping. These studies identified molecular markers associated with the selected characters, preliminarily screened excellent progeny, and provided important basic genetic resources for chrysanthemum-breeding programs. However, the reported linkage maps are not integrated and are mainly based on traditional gel-based SRAP, RAPD, ISSR, AFLP, and SSR markers, which present a low density and are difficult to transfer between laboratories. Although several major-effect QTLs have been detected for these traits, it is currently difficult to identify the chromosomal locations of these QTLs and to validate these QTLs in other populations; therefore, it is impossible to find the genes underlying the desired traits.

High-density linkage maps constructed with many DNA markers provide insights into the chromosomal composition of plants, which are important for techniques used in genetic studies and breeding programs such as MAS, QTL mapping, and map-based gene cloning^84^. Currently, advanced high-throughput genotyping technology offers an opportunity to develop large-scale sequenced SNP markers and construct high-density genetic maps in ornamentals such as sunflower^85^ and the Chinese endemic *Dendrobium*^86^. These SNPs are mostly biallelic and can therefore distinguish only two alleles; however, there are allele dosage effects at a single locus in polyploids. Tetraploid rose is the species in which the mode of inheritance has been the most investigated and presents the highest-density map construction among polyploid ornamental species by far^87,88^. Koning-Boucoiran et al. speculated that the characteristics of tetraploid rose are more in line with tetrasomic inheritance or a mixture of both modes of inheritance than with disomic inheritance according to the segregation patterns of molecular markers in a tetraploid-mapping population of 184 genotypes^89^. Bourke et al. found that partial preferential chromosome pairing is genotype dependent in tetraploid rose and confirmed the inferred differences in pairing behavior between chromosomes by examining repulsion-phase linkage estimates^90^. Recently, convincing evidence for the hexasomic inheritance of cultivated chrysanthemums was provided based on the analysis of a 183-k SNP genotyping array generated from RNA sequencing data, and the authors noted that chrysanthemums were more likely to be autohexaploid than allohexaploid^91^. Based on SNPs and this assumption, Van Geest et al.^92^ published the first integrated ultradense linkage map of a biparental population, including 406 hexaploid individuals, using polymapR, which was developed for the genetic linkage analysis of outcrossing autopolyploids, i.e., triploids, tetraploids, and hexaploids, based on marker dosage. This map contained nine chromosomal LGs and covered 752.1 cM, with an average of 3368 markers per LG. Based on the linkage map, several QTLs controlling flower color, flowering time, ray floret number, and disc floret degreening were identified through multiallelic QTL analyses. This research is a critical step forward for linkage analysis in chrysanthemum. However, whether cultivated chrysanthemum is an allopolyploid or autopolyploid remains controversial. Only by answering these questions can researchers choose appropriate mapping strategies and software tools. A lack of complete genome information remains the largest bottleneck, making further fine mapping almost impossible.

#### Genome-wide association mapping

Genome-wide association studies (GWASs) are another effective approach for connecting phenotypes with genotypes in plants when information on population structure and linkage disequilibrium is available^93^. Successful examples of GWASs in crop species have been recently reviewed^94^. In the case of chrysanthemum, GWASs have been used to explore inheritance patterns and identify favorable alleles for several ornamental characteristics and resistance traits, including plant architecture traits and inflorescence traits^95^, waterlogging tolerance^96^, aphid resistance^97^, and drought tolerance^98^, based on 707 conventional gel-based SRAP, start codon-targeted (SCoT), and SSR marker loci. These studies successfully identified several favorable alleles associated with the traits of interest and screened elite germplasm resources as potential donors for chrysanthemum breeding. However, little information about the genome or transcriptome of chrysanthemum is available due to the limited number of markers, low marker reliability, high cost, and high labor intensity associated with the currently used conventional marker types. Nevertheless, the advent of NGS technologies has created new opportunities for high-throughput marker development and more efficient marker screens in unsequenced species. Chong et al.^9^ first attempted to develop SNP markers utilizing specific length- amplified fragment sequencing (SLAF-seq) technology in a panel of 199 chrysanthemum accessions and detected 97 SNPs associated with important horticultural traits via a GWAS. Furthermore, six candidate genes harboring seven of the 97 associated SNPs were predicted by aligning the SLAF sequences to the chrysanthemum transcriptome database. This study represents a large breakthrough in integrating phenotypic and genetic data toward the discovery of candidate genes in chrysanthemum. Several candidate genes related to waterlogging tolerance and inflorescence-related traits were recently identified based on the above-mentioned SNP sets in a similar way^99,100^. Using another method, Klie et al.^101^ were able to identify 11 markers significantly associated with four strigolactone pathway genes regulating shoot branching in chrysanthemum by utilizing the candidate gene approach, which also provided insight into some potential candidate genes.

Notably, markers should be technically simple enough to be effectively applied in the selection of superior progeny, in contrast to those used in a research laboratory^12^. Su et al.^99^ transferred a major SNP locus that cosegregated with waterlogging tolerance in chrysanthemum into a superior PCR-based derived cleaved amplified polymorphism sequence (dCAPS) marker with a moderately high accuracy of 78.9%, which was then verified in 52 cultivars or progeny. Chong et al.^100^ developed two dCAPS markers correlated with flowering time and capitulum diameter in chrysanthemum. These dCAPS markers present potential value for practical application in chrysanthemum MAS breeding. SNPs can also be converted to other available markers, such as high-resolution melting (HRM)^102^ and kompetitive allele-specific (KASP)^103^ markers, and be used for amplicon sequencing^104^ in polyploid plants. These technologies will provide new and powerful tools for future chrysanthemum breeding.

#### Bulked segregant analysis

The bulked segregant analysis (BSA) strategy proposed by Michelmore enables researchers to simply and quickly identify markers, specific genes, or genomic regions associated with a target trait using two DNA bulks consisting of individuals exhibiting contrasting extreme phenotypes^105^. In chrysanthemum, several studies on different horticultural traits have used BSA methods. Wang et al.^80^ detected two SSR markers linked to aphid resistance; Klie et al.^101^ found two AFLP markers showing significant allele frequency differences in bulks with a low or high degree of shoot branching; Sang et al.^106^ identified one AFLP marker linked to white rust and converted it into a SCAR marker, which was further verified in a pseudo-F_1_ testcross and had the potential to be used to select white-rust-resistant plants in chrysanthemum-breeding programs. Recently, several studies have reported that combining the BSA strategy with high-throughput sequencing technology provides a new opportunity to accelerate the screening of candidate genes associated with important traits in ornamental plants^107^. However, the NGS-assisted BSA approach is usually applied to species in which genome information is available at least for closely related plant species. Recently, a modification of BSA that couples RNA-seq with BSA, termed bulked segregant RNA-seq (BSR-seq), was proposed to allow the correlation of gene expression and markers with phenotypes^108^. BSR-seq combines the superior features of RNA-seq and BSA and enables the establishment of a reference sequence by *de novo* transcriptome assembly for variation calling in nonmodel organisms; this technique has been successfully applied in species with complex genomes such as wheat^109,110^ and peony^111^.

#### Remarks on MAS

MAS is an effective tool in modern plant breeding. However, the successful application of MAS in practical breeding thus far has been limited to simple traits with monogenic or oligogenic inheritance in crops such as rice, soybean, and maize^112^. The application of MAS has several technical and logistical prerequisites, such as marker type involved, the inheritance schema of the target traits, the number of target genes in genomic regions, and their distance from the flanking markers^113^. Although various DNA markers are now routinely used in basic quantitative genetic studies of chrysanthemum, comparatively few practical applications of these molecular markers in the genetic improvement of horticultural traits have been reported. The main problems lie in the slow process of MAS in chrysanthemum: (1) the population sizes and marker numbers are too small for accurate high-resolution mapping, especially in linkage analysis; (2) the detected QTLs are mostly minor-effect and environment-specific QTLs with no validation, which are not robust enough for selecting phenotypes and performing positional gene cloning; (3) it is difficult to determine the location in the genome of these detected associations or candidate genes due to the lack of complete genome information; (4) all QTL mapping and BSA studies are currently based on F_1_ populations. The construction of permanent populations such as doubled haploids (DHs), near-isogenic lines (NILs), or recombinant inbred lines (RILs) in heterozygous chrysanthemum is not possible, making the genetic dissection and fine mapping of traits difficult.

To overcome these hurdles, a large population should be used in future studies to trace recombination events. Large-scale, high-throughput, low-cost SNPs are already available in chrysanthemums due to advances in NGS technology. However, the quantity and quality of phenotyping are becoming important determinants of the accuracy of genetic mapping, and thus, of the power of the resultant MAS, particularly for complex quantitative traits, because breeders must continue phenotypic evaluation and selection of the lines during the process of breeding. Plants exhibit considerable changes in gene expression during different developmental stages and when exposed to a range of environmental stresses^114^. Hence, the collection of phenotypic data at different stages for dynamic mapping is also an essential prerequisite to achieve breeding goals. Therefore, high-throughput phenotyping techniques for the collection of precise phenotypic data are urgently needed. In addition, closer cooperation is needed between breeders and molecular geneticists to effectively convert the markers from the laboratory to the field.

## Omics technologies

### Genomics

Genomic research has a vast capacity to accelerate the breeding process and presents applications for genetic improvement such as MAS and gene pyramiding. The genomic information of cultivated chrysanthemum has not been reported, mainly due to its unknown origin, heterozygosity, extremely large genome size (9.4 Gb, http://www.etnobiofic.cat/gsad_v2/), and high repeat content. Early on, bacterial artificial chromosome (BAC) libraries were widely used for whole-genome sequencing (WGS). However, this approach consisted of a laborious process of constructing a physical map composed of many BAC clones^16^. The *de novo* assembly of large genomes with large numbers of repetitive sequences and a high degree of heterozygosity based on second-generation sequencing platforms remains a challenge due to insufficient read length^115^. However, the emergence of third-generation sequencing platform technologies represented by single-molecule real-time sequencing (SMRT-seq) technology from the Pacific Biosciences (PacBio) platform and nanopore single-molecule sequencing technology from Oxford Nanopore designed to generate long reads, along with the rapid development of bioinformatics, has greatly accelerated sequence assembly and enabled the generation of high-quality assemblies^116^. Recently, the genome sequences of several Asteraceae species, including *Cynara scolymus* L. (2n = 2x = 34; 1.08 Gb)^117^, *Helianthus annuus* L. (2n = 2x = 34; 3.6 Gb)^118^, *Lactuca sativa* L. (2n = 2x = 18; 2.38 Gb)^119^, and *Artemisia annua* (2n = 2x = 18; 1.74 Gb)^120^, have been revealed due to advances in sequencing technologies. More recently, the genome information of two diploid *Chrysanthemum* species, *C. nankingense*^121^ and *C. seticuspe*^122^, which are potential progenitors or model species of domesticated chrysanthemums, was published. The genome assembly of *C. nankingense* using 105.2 Gb of Oxford Nanopore long-read data combined with 362.3 Gb of Illumina short reads, representing ~82% of the estimated genome size, yielded 24,051 sequence contigs with a total size of 2.53 Gb and an N50 of 130.7 kb. A total of 56,870 protein-coding genes were identified, with 69.6% of the assembly annotated as repetitive elements. This research showed that *C. nankingense* genome evolution was driven by bursts of repetitive element expansion and whole-genome duplication (WGD) events. In addition, the expansion of gene families by duplication events may lead directly to variation in the ornamental and medicinal characteristics of chrysanthemum. The genome assembly of *C. seticuspe* using the Illumina sequencing platform produced 2.72 Gb of sequences consisting of 354,212 scaffolds with an N50 length of 44.7 kb, covering ~89.0% of the estimated genome size. Based on the assembled genome, a total of 71,057 protein-coding genes were recovered, and a CEN-like gene potentially related to flowering was newly identified. SNP identification and annotation performed by mapping the transcriptome data of six chrysanthemum varieties onto the *C. seticuspe* genome demonstrated that the *C. seticuspe* genome is applicable to genetic analysis in cultivated chrysanthemums. The published genome sequence information of the two diploid chrysanthemums will undoubtedly offer new resources and guidance for elucidating the origin and evolution of cultivated chrysanthemums. In addition, the emergence and constant improvement of Hi-C technology provide an opportunity to assemble the genomes of complex species at the chromosome level, making it a promising prospect for the future WGS of cultivated chrysanthemum.

### Transcriptomics

Transcriptomics is the study of the transcriptome, specifically characterizing and quantifying the complete set of RNAs present in a specific organ, tissue, or cell in a given organism using high-throughput methods^123^. In contrast to the static entities in the genome, the transcriptome is dynamic in its expression. Recently, the continual refinement of mRNA sequencing (RNA-seq) and *de novo* sequencing and assembly technology has allowed an increasing number of laboratories to characterize the transcriptomes of nonmodel species with large genomes. Moreover, several public data repositories now make it possible to exchange transcriptome data among research communities. In chrysanthemum, transcriptome sequencing is considered an effective method for elucidating the gene regulation mechanisms and networks controlling various complex biological processes and performing comparative transcriptomics studies (Table 2). In addition, the massive quantity of transcript sequences, including differentially expressed genes (DEGs), available for diverse genotypes can considerably aid in discovering interesting genes and developing a vast number of SSR and SNP markers. This in turn has propelled the large-scale mining and evaluation of novel functional allelic variation diversity levels for transgenic breeding and MAS applications.

**Table 2:** Recent published transcriptomic studies in chrysanthemum

Category	Material	Plant organ/developmental stage	Study objective	Methodology	Reference
Plant growth and development	*C. lavandulifolium*	Leaves in seedling and visible bud stage	Flowering	RNA-seq	^125^
	‘Fenditan’	Vegetative buds, floral buds, and buds	Flower development	RNA-seq	^126^
	‘Jinba’	Whorl petals at four developmental stages	Petal growth	RNA-seq	^127^
Stress resistance	‘Fall Color’	Leaves and roots	Drought stress	RNA-seq	^128^
	‘Jinba’	Leaves and roots	Salt stress	RNA-seq	^129^
	‘Jinba’	Seedlings	Cold stress	RNA-seq	^130^
	*C. nankingense*	Leaf	Heat stress	RNA-seq	^131^
	‘Nannongxuefeng’, ‘Qinglu'	Roots	Waterlogging stress	RNA-seq	^132^
	‘Nannongxunzhang’	Leaf	Aphid stress	miRNAs	^134^
	‘Zaoyihong’	Leaf	Disease	RNA-seq	^135^
	‘Shinm’	Leaf	RNA viruses	Microarray	^136^
	‘C029’, ‘C008’	Leaf	White rust	RNA-seq	^137^
Secondary metabolism	‘Purple Reagan’	Ray florets at five developmental stages	Anthocyanin biosynthesis	RNA-seq	^143^
	‘Feeling White’, ‘Feeling Green’, and ‘Feeling Green Dark’	Ray florets	Chlorophyll metabolism in petal	Microarray	^144^
	‘Chuju’	Flowers at four developmental stages	Flavonoid biosynthesis	RNA-seq	^145^

#### Plant growth and development

Plant growth and development is a complicated hierarchical system that is dynamically controlled by a network composed of various genes. Flowering is a complex developmental process in plants during which morphological and physiological changes affecting several external and internal factors occur^124^. By performing RNA-seq, Wang et al.^125^ identified several unigenes related to photoperiod, vernalization, gibberellin, and autonomous pathways that exhibited dramatically different expression levels between the seedling stage and the visible bud stage in *C. lavandulifolium*. Many TF genes, such as MIKC type, YABBY, and SBP, were selected as candidate genes for flowering in chrysanthemums. Petal size directly determines the appreciation value of chrysanthemum^126^. Wang et al.^127^ performed an RNA-seq analysis by collecting data at different petal development stages in the chrysanthemum cv. ‘Jinba’ and identified MADS, TCP, and bHLH as the three primary types of TFs associated with flower development. Further studies showed that *TCP4* and *TCP9* may function as positive regulators of chrysanthemum petal growth. Novel flower shape is of great importance for marketing in ornamental plants. A comparative transcriptome analysis between the florets and leaves revealed several key DEGs involved in flower development, flower organ differentiation, and anthocyanin biosynthesis^126^.

#### Stress resistance

Mining crucial genetic resources controlling resistance characters will greatly contribute to breeding programs. Transcriptome-sequencing technology has been widely used for the identification of major regulatory genes and gene networks controlling responses to several abiotic stresses (i.e., drought^128^, salt^129^, cold^130^, heat^131^, and waterlogging^132^). MicroRNAs (miRNAs), which are ~22 nt in size, are the most abundant class of endogenous noncoding small RNAs (smRNAs) in plants and play important roles in plant biological processes^133^. Chrysanthemums are easily attacked by aphids throughout the growing season, seriously affecting their growth and ornamental value. Xia et al.^134^ quantified the abundance of miRNAs induced by aphid infestation in chrysanthemum leaves and identified miR159a, miR160a, and miR393a as the most likely miRNAs to respond to aphid infestation in chrysanthemum. In addition, pathogens such as viruses, viroids, fungi, and phytoplasma seriously reduce the production of chrysanthemums. Transcriptome-sequencing technology has also been used to examine disease resistance to chrysanthemum black spot disease^135^, several viral diseases^136^, and white-rust disease^137^. However, all the reported transcriptome studies of chrysanthemum have been based on microarray or second-generation sequencing platforms producing reads with lengths of <400 bp. Due to this read length limitation, more chimeras are produced in the process of transcript assembly, and complete transcript information cannot be accurately obtained; this problem greatly reduces the accuracy of quantitative expression, alternative splicing, and gene fusion studies. The PacBio SMRT-seq technology platform has introduced powerful new tools to help solve this problem. Although the longer SMRT-seq reads are less accurate, they can be modified by more accurate data from second-generation sequencing^116,138^. Recently, Zhao et al.^139^ performed a comparative transcriptome study of salt stress using RNA-seq and SMRT-seq to improve the quality and accuracy of the results. As a result, many DEGs associated with signal transduction, the biofilm system, the antioxidant system, and the osmotic regulation system, such as MAPK, ACOT, SOD, CAT, PMP, and P5CR, were identified. The responses of plants to stresses and the acquisition of stress resistance are regulated by a complex system of interacting signals and are susceptible to some environmental factors. Although a wide range of genes or TFs involved in different signaling pathway responses to stresses have been identified with transcriptome-sequencing technology in chrysanthemum, these studies were specific to a particular stress, and thus, contribute less to a comprehensive understanding of this complex system. Therefore, designing a rational set of experiments to investigate common genes or pathway responses to multiple stresses would provide breeders with an opportunity to improve the synthetic resistance of chrysanthemum by developing gene-specific molecular markers or pursuing transgenic breeding.

#### Secondary metabolism

Plants can synthesize various low-molecular-weight organic compounds, termed secondary metabolites, which generally possess attractive biological activities and are useful as pharmaceuticals, flavorings, fragrances, and insecticides^140^. Therefore, secondary metabolites are important targets for breeding programs, and their biosynthetic pathways are a topic of study. Flower color is determined by several metabolites including anthocyanins, carotenoids, and chlorophylls and influences the commercial value of chrysanthemum cultivars^141^. Light is a major external factor affecting anthocyanin biosynthesis^142^. Five libraries were designed for transcriptomic analyses to investigate the molecular mechanisms of light-induced anthocyanin biosynthesis, and the results revealed three *CmMYB* genes and one *CmbHLH* gene as primary TFs that might regulate anthocyanin biosynthesis under light conditions^143^. Recently, increasing numbers of green-flowered chrysanthemum cultivars have appeared on the flower market and have been greatly admired by their purchasers. Chlorophyll accumulation is the main determinant of the green flower color, and the chlorophyll content decreases as petal development progresses^144^. The expression levels of chlorophyll-related genes in white- and green-flowered chrysanthemum cultivars were compared by Ohmiya et al.^144^ using microarray analyses. Among these genes, *STAY GREEN* (*SRG*), which encodes Mg dechelatase, a key enzyme controlling chlorophyll degradation, showed markedly higher levels in petals. Chrysanthemums have also been used in teas after special processing throughout history, and the development of new tea cultivars with improved medicinal and nutritional quality is a new direction for chrysanthemum breeding. Yue et al.^145^ performed a transcriptome analysis of ‘Chuju’ chrysanthemum, a well-known medicinal species. A MYB-bHLH-WD40 (MBW) complex was found to tightly regulate flavonoid biosynthesis along with flower development. A coexpression network analysis indicated that different members of the MBW complex might be subject to different positive or negative regulatory mechanisms. Several regulatory and structural candidate genes possibly involved in flavonoid biosynthesis were detected and characterized, and these genes will provide valuable genetic resources for studies of nutrition improvement and transgenic breeding in chrysanthemum.

### Proteomics

Proteomic approaches have been successfully used in several sequenced plants, such as rice and *Arabidopsis*, to study diverse bioprocesses and environmental adaptations^146,147^. Ornamental chrysanthemums are traditionally cultivated by vegetative stem cutting, after which regeneration occurs via adventitious roots; therefore, the investigation of the molecular mechanisms underlying adventitious root formation is of particular significance in chrysanthemum breeding. Using the classical two-dimensional electrophoresis (2-DE) method, Liu et al.^148^ successfully identified 69 differentially accumulated protein spots between cut chrysanthemum bases of different ages. Most of the detected proteins were linked to carbohydrates, photosynthesis, energy metabolism, and cell structure, and the auxin-induced proteins PCNT115 and CmACO were tightly correlated with adventitious root formation, as supported by western blot experiments. A comparative proteomic analysis of postharvest medicinal chrysanthemum flowers under normal or UV-B radiation conditions allowed the detection of 43 differentially accumulated protein spots, some of which were identified as participating in photosynthesis, respiration, and defense^149^. The primary drawbacks of 2-DE are its low repeatability and resolution. To overcome these limitations, novel protein identification and quantification techniques with high accuracy, such as the isobaric tags for relative and absolute quantitation (iTRAQ) method and mass spectrometry (MS), have been developed^150^.

A comparative proteomic analysis was performed using two contrasting chrysanthemum varieties that were either subjected to 6 h of heat stress or not using iTRAQ combined with tandem MS and multidimensional liquid chromatography technology^151^. The research identified 250 differentially expressed proteins, 43 of which were involved in photosynthesis, metabolic processes, oxidation-reduction processes, and transport. Subsequently, iTRAQ was successfully applied in proteomic studies of embryo abortion^152,153^ and pollen abortion in chrysanthemum^154^. Although several protein-related studies have been published for chrysanthemum, the number lags far behind that for model plants and is significantly lower than the number of transcriptomics studies.

### Metabolomics

Metabolomics is a postgenomic technology that seeks to provide a comprehensive profile of all the metabolites (estimated to be at least 200,000) present in a typical plant and represents an important branch of systems biology^155^. Miyazawa and Hisama^156^ isolated and identified the antimutagenic metabolites in the flower heads of chrysanthemum by electron-impact (EI)-MS, infrared (IR) spectroscopy, and nuclear magnetic resonance (NMR) spectrometry. The analysis concluded that the flavonoids acacetin, apigenin, and luteolin were primary compounds in chrysanthemum and may be potent biological antimutagens that are useful as cancer chemopreventive agents. A standardized profiling method, liquid chromatography-diode array detection-electrospray ionization/MS (LC-DAD-ESI/MS), was used to successfully detect some flavonoids and caffeic acid derivatives in the flowers of chrysanthemum, clarifying the biological activity of chrysanthemum and its human health benefits^11^. Liu et al.^157^ proposed a green method for the characterization and quantitation of chrysanthemum metabolites by pressurized hot-water extraction coupled with high-performance LC (HPLC)/UV radiation. Chrysanthemum is a typical short-day plant with photoperiod-induced flowering. Kjaer et al.^158^ reported a larger leaf area of chrysanthemum plants under long-day conditions than under short-day conditions and short-day conditions with irregular night interruption conditions. Further research indicated that photoperiodic variation changed the leaf metabolic profile, but only carbohydrates, citrate, and some amino acids showed a shift in the diurnal pattern. Flowers produce various particular metabolites, such as fragrant volatiles, to attract pollinators, initiate or inhibit signaling cascades, and protect against herbivores or pathogens^159^. The types of metabolites vary among different species, and even different genotypes of a certain species may exhibit a wide range of these substances^160^. An effective multicomponent quantification fingerprinting method combining HPLC, DAD, and ESI tandem/MS (HPLC-DAD-ESI/MS) was established and shown to simultaneously determine several caffeoylquinic acid derivatives and flavonoids in chrysanthemum cultivars^161^. The qualitative and quantitative analysis of the major volatile compounds produced by flowers among different chrysanthemum materials determined by gas chromatography (GC)-MS showed that monoterpenoids and oxygenated monoterpenoids were the major organic components^162^, and that more abundant volatiles could be identified in chrysanthemum cultivars than in wild plants. Developing new cultivars with a long vase life and long storability is challenging for chrysanthemum breeders. van Geest et al.^163^ were able to show metabolic profile differences under carbohydrate starvation using NMR spectroscopy and high-performance anion-exchange chromatography (HPAEC), revealing that the camphor, phenylpropanoid, and flavonoid contents of the plants were responsible for the genotype-specific profiles, but the carbohydrate contents could not explain all the variation in degreening sensitivity. A metabolomic strategy based on combined headspace-GC-MS (HSGC-MS) and ultra-HPLC (UHPLC)-MS data sets was first proposed by Zhang et al.^164^ to analyze the volatile and nonvolatile substances present in chrysanthemum, respectively. More recently, a number of differential primary metabolite profiles were identified in the leaves and roots of wild-type and transgenic *CmPht1;2* plants, revealing that overexpression of *CmPht1;2* might improve low-phosphorus stress via metabolic adaptation in chrysanthemum^165^.

Overall, the metabolome is influenced by genotype, tissue, developmental stage, and the plant’s ecological habitat. Metabolomics is an important extended omics layer that can provide chemical evidence allowing us to understand the phenomena occurring in cells or tissues under certain conditions^18^. Currently, metabolome profiling can be used to evaluate metabolic phenotypes and to identify metabolite QTLs (mQTLs) in segregating or natural populations, which contributes to the breeding of traits associated with the production of functionally useful metabolites in crops. To date, several fairly mature technologies or sophisticated combinations of these tools have been employed in metabolomic analyses of chrysanthemum and have generated very large amounts of data. However, no studies of mQTLs have been reported. Therefore, developing specific software to improve the capacity to recognize different metabolites and combining the identified metabolites with genetic studies whose results can be utilized in actual chrysanthemum-breeding programs are the main challenges faced by researchers in related fields.

### Microbiomics

Microbiomics is a discipline that applies omics technologies to plant microbial communities. The rhizosphere microbiome, which includes bacterial and fungal communities, directly affects plant immunity, pathogen abundance, nutrient acquisition, and stress tolerance, among other characteristics. Plants live in association with diverse microorganisms, and plant-microbe interactions harbor a vast capacity to change invasive growth characteristics^166,167^. The study of plant microbiomes depends on both laboratory culture techniques and species identification based on PCR amplification followed by sequencing, microarray analysis, terminal restriction fragment length polymorphism (TRFLP) analysis, or various electrophoretic procedures, including denaturing gradient gel electrophoresis (DGGE) and temperature gradient gel electrophoresis (TGGE)^168^. The abundance and diversity of many bacterial and fungal communities have been investigated via DGGE in chrysanthemum^169,170^. As observed for most crop species, the production of chrysanthemum is decreasing dramatically because of serious obstacles to continuous cropping. Chrysanthemums are easily infected by *Fusarium* wilt, which is regarded as the most serious soil-borne facultative pathogen, in long-term monoculture^171^. Control of this disease is currently achieved with chemical fungicides and soil fumigation, but these measures might negatively influence the environment and soil ecosystem. A biological control method was proposed by Liu et al.^172^ in which chlamydospores of *Phanerochaete chrysosporium* were inoculated into the chrysanthemum rhizosphere to reduce this continuous-cropping obstacle by changing the microbial community, phenolic acid content, and enzyme activity. In addition, Cipriano and Freitas^173^ demonstrated that two *Pseudomonas putida* strains can be used as beneficial microorganisms and present great potential to safely and effectively promote plant growth and flowering under field conditions. Several publications have shown the usefulness of NGS technologies for revealing microorganisms that live in association with plants^174,175^. However, there has been little research on the microbiomes of ornamental species, including chrysanthemum, using NGS.

## Conclusions and perspectives

### Problems associated with molecular breeding in chrysanthemum

Although substantial progress has been made in transgenic breeding and MAS technologies for horticultural trait improvement in chrysanthemum, as mentioned in this review, many technical and scientific challenges that prevent the application of these technologies in actual breeding programs remain. First, the horticultural industry is paying attention to improved safety of products and environmental protection. To achieve these goals and remain competitive, further technical innovations and scientific transgenic plant safety evaluation systems are needed to bring transgenic chrysanthemums to the market. Furthermore, the application of new gene-editing technologies, such as the use of the CRISPR/Cas9 system, zinc finger nucleases (ZFNs), and transcription activator-like effector nucleases (TALENs), has enormous potential to benefit the ornamental horticulture industry by supporting targeted genomic modifications. However, the development of specific experimental and analytical methods for polyploids is urgently required. The rapid development of molecular marker technology, especially the availability of large-scale SNPs, is a revolutionary change for chrysanthemum. Diploid genome sequences can be used as references for genetic and genomic analysis in polyploid species. Currently, the release of the genomes of the two diploids, *C. nankingense* and *C. seticuspe*, as reference plants, is shedding light on the complex nature of the hexaploid-cultivated chrysanthemum. Hence, the development sequence-based markers such as SNPs, InDels, and structural variants (SVs) that can be used for linkage and association-mapping studies in a large population is becoming possible. However, to achieve this goal, the genetic relations between the two diploids and cultivated chrysanthemums should first be investigated to guarantee a sufficient mapping ratio. Moreover, developing ‘breeder-friendly’ markers that could reduce the cost and time of high-throughput genotyping while lowering error rates will certainly allow the screening of allele composition to be incorporated into breeding programs, for example, through the use of gel-free KASP markers or the conversion of nonbreeder-friendly markers to other types of ‘breeder-friendly’ markers (e.g., RFLP to STS, RAPD to SCAR).

### Multiomic integration facilitates the understanding of biological systems

As the development of NGS technologies and the emergence of multiple omics platforms and technical innovations allow the constant production of a wealth of genomic, transcriptomic, proteomic, metabolomic, and microbiomic data, we are clearly approaching the era of ‘big biological data’. In recent years, breakthroughs have been made in the application of these omics technologies in chrysanthemum, but such applications are still in their infancy, lagging far behind those for model plants and some crop plants, and they are used mainly in transcriptomic studies. As the preceding overview of this process shows, the lack of full-genome sequences is undoubtedly the foremost limitation to scientific studies of chrysanthemum. The generation of the full-genome sequences of the two diploid *Chrysanthemum* species is expected to contribute to the identification of genes of interest and the future genome assembly of cultivated chrysanthemum. However, bioinformatics tools and algorithms that can be used to obtain reliable genome sequences represent the most urgent problem. Moreover, phenotyping large populations across developmental stages and in response to environmental variables is now the most challenging task of the breeding process^176^. The introduction of phenomics offers the possibility of revealing the genetic mechanisms underlying some previously intractable scientific problems in detail by using recently developed advanced sensors, cameras, computers, robotics, and machine vision and image analysis tools^177^. Integrating and interpreting this global information composed of different hierarchical multiomic data sets in a synthetic manner will help provide a comprehensive understanding of the dynamic molecular regulatory mechanisms and gene networks that are active in plants^178^. Multiomic integration is expected to accelerate the plant-breeding process. To date, only a few attempts have been made to combine transcriptomic and proteomic data in chrysanthemum^152–154^. Thus, developing cost-effective high-throughput phenotyping platforms, establishing sustainable and integrative omics data management systems, developing bioinformatics tools and more efficient algorithms, and building specialized databases that are accessible on the Internet to satisfy the ever-increasing needs for data size and analysis are of great urgency for both researchers and breeders of chrysanthemum.

### Prospects of chrysanthemum breeding

**Fig. 2:**
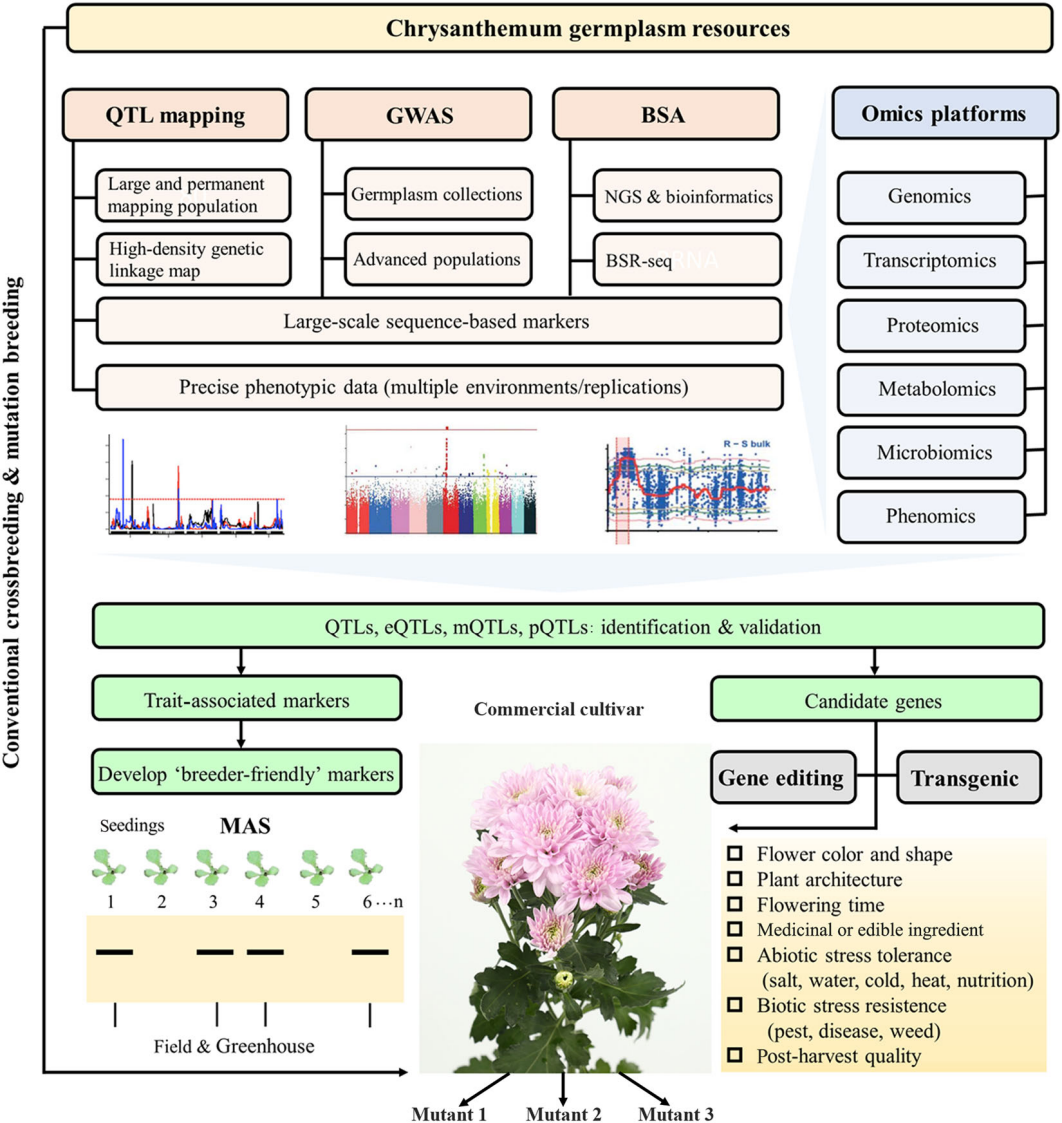
A hypothetical comprehensive breeding scheme for chrysanthemum integrating conventional with modern breeding strategies

With the rising demand for chrysanthemum, the development of new attractive cultivars with comprehensive resistance to various environmental stresses is urgently needed. Molecular and omics methods are expected to expedite the breeding process compared with conventional methods, which remain the mainstay of practical breeding programs for hexaploid chrysanthemum. The integration of genomic and other omics data with genetic and phenotypic data contributes to the identification of genes/pathways associated with important traits^179^. In view of the respective advantages and bottlenecks of these breeding strategies, as discussed above, we propose a comprehensive chrysanthemum-breeding scheme, which is shown in Fig. 2. Selecting suitable parents or populations with desired traits from the abundant germplasm resources of chrysanthemum, which are the materials used for genetic studies, is the fundamental basis of breeding programs. Large-scale sequence-based markers, such as SNPs and InDels, and precise phenotypic data could be easily obtained through multiomic technologies and provide a crucial basis for the application of GWAS and QTL-mapping analysis to locate genes of interest and linkage markers associated with the target traits. The validated QTLs/markers could be used directly or transformed into ‘breeder-friendly’ markers to be effectively used in MAS, expediting the breeding process. Notably, transcript, protein, and metabolite profiles may serve as phenotypic information in linkage-mapping populations or natural populations to detect loci controlling their expression levels; such analyses are known as eQTL/eGWAS, pQTL/pGWAS, and mQTL/mGWAS, respectively. These methods have been used for several species with continuous improvement^179–182^. Moreover, the key candidate genes identified by genetic studies or omics methods could be utilized through transgenic or gene-editing technologies. Finally, if a genotype is ready to become a commercial cultivar, a novel flower color and shape, similar to other desired traits, could be easily generated through mutation breeding. Overall, the exploration of feasible methods for employing these new technologies is still in the initial stage for chrysanthemum. Despite the restrictions discussed in this article, the combination of modern breeding strategies with conventional methods would clearly be a superior approach for improving major horticultural characteristics, incorporating multiple characters into a single cultivar and expanding the usage of chrysanthemum in the floriculture industry.
